# The impact of a mindfulness bracelet on emotional affect in medical students: a prospective cohort study

**DOI:** 10.1186/s12909-022-03935-1

**Published:** 2022-12-10

**Authors:** Michael LoBasso, Ian A. Jones, Johanna Shapiro, Soheil Saadat, Alisa Wray

**Affiliations:** 1 University of California, Los Angeles, USA; 2 University of Washington, Seattle, USA; 3 University of California, Irvine, USA

**Keywords:** Medical students, Medical school, Mindfulness, Emotional affect, Positive affect, Stress

## Abstract

**Background:**

There is concern that negative changes in emotional outlook among medical students may impair the behavior of students, diminish learning, and ultimately affect patient care. Although most medical students begin their professional education with idealism, enthusiasm, and attention to humanity, they often have difficulty balancing their happiness with social and professional responsibilities. The following study aimed to determine if a simple mindfulness reminder (in the form of a bracelet) will impact emotional affect in first-year medical students. The second aim is to better understand the changes in emotional affect as students start medical school.

**Methods:**

First year medical students were invited to participate at the start of the academic year. Baseline survey data and demographic data were obtained prior to participants being given the mindfulness bracelet and a standardized presentation explaining its purpose. Follow-up surveys were obtained at one- and two-month intervals. Statistical analysis was based on sum score of the survey. Change of sum score over time was tested by using repeated measurement analysis.

**Results:**

Data collection included 104 students at the initial distribution of the survey. Follow-up surveys were obtained from 78 and 69 students at the first- and second-month mark, respectively. No significant associations were detected between frequency of mindfulness bracelet usage and emotional affect. However, there was a significant decrease in positive affect over the first month of medical school (*p* < 0.01), followed by a significant recovery in positive affect in the second month of medical school (p < 0.01). Demographic data did not reveal statistically significant differences among demographic groups and progression of emotional affect.

**Conclusions:**

Although the mindfulness bracelet intervention did not yield significant improvement in emotional affect, our results are consistent with other studies demonstrating that the first year of medical school negatively impacts the emotional outlook of students. Future studies are needed to explore practical interventions and to better understand and address the negative effect that early medical school education has on student’s emotional state.

## Background

Medical students face a rigorous workload, including the newly experienced pressures of the clinical environment. Although most medical students begin their professional education with idealism, enthusiasm, and attention to humanity, they often have difficulty balancing their happiness with social and professional responsibilities [1]. There is concern that negative changes in emotional outlook among medical students may impair the behavior of students, diminish learning, and ultimately affect patient care during schooling and in practice [2]. Multiple studies demonstrate that medical students experience higher levels of psychological distress including depression, anxiety, and suicidal ideation than non-medical students [3, 4]. Among medical students, the prevalence of anxiety has been shown to be between 40 and 79% [5] and a recent meta-analysis demonstrates the prevalence of depression and suicidal ideation to be 27.2 and 11.1%, respectively [6]. Student distress contributes to cynicism and subsequently affects students’ care for patients, relationships with faculty and ultimately the culture of the medical profession [7]. There is justified concern that psychological distress during medical school will continue into professional roles and increase the likelihood of professional burnout [8]. Stress impacts emotional affect, which is defined as the mental counterpart of internal bodily representations associated with emotions and dispositions [9].

Over the past two decades there has been increasing interest in mindfulness as a form of clinical intervention, with growing evidence for its positive impact on well-being [10, 11]. Mindfulness has been described as the awareness and non-judgmental acceptance of one’s moment-to-moment experience to combat common forms of psychological distress - namely anxiety, fear, anger, rumination, etc. [11, 12]. Incorporation of formal mindfulness practice including meditation and journaling has demonstrated significant positive changes in self-reported empathy and kindness among first year medical students [13]. Interestingly, informal mindfulness practice, which refers to mindfulness outside of traditional mediums, whereby one finds mindfulness in their daily activities (e.g. eating, walking, or performing daily chores) has been correlated with improved psychological wellbeing compared to formal mindfulness practice [8]. The role of a simple, cost-effective reminder to participate in these informal mindfulness techniques has yet to be studied. The primary aim of this study was to determine if a simple mindfulness reminder (in the form of a bracelet) could improve the emotional affect of medical students, as measured by the Patient Reported Outcomes Measurement Information System (PROMIS) Positive Affect short form v1.0 survey. The secondary aim of this study was to measure the trends in emotional affect as students begin their first year of medical education.

## Methods

### Data collection

All study activities were conducted at the University of California, Irvine School of Medicine. All first-year medical students were invited to participate. In total 3 surveys were obtained in this study. The baseline survey, which included both demographic questions and PROMIS questionnaire, was collected on August 10th, 2020. Follow-up surveys, which included a question regarding how often students used their mindfulness reminder and PROMIS questionnaire, were obtained on September 22nd, 2020 and October 16th, 2020. These time points correspond to the first, second, and third months of medical school. At the time of the study, first year medical students were engaging in didactic education, completing performance exams, and participating in standardized clinical encounters. The distribution of the initial survey was followed by a standardized 10-minute presentation outlining the purpose of the mindfulness bracelet to serve as a reminder to intentionally practice informal mindfulness techniques. These informal mindfulness techniques were explicitly stated as pausing to non-judgmentally acknowledge one’s moment-to-moment experience in their daily life, be present in the activity or task at hand, and express a feeling of gratitude [13]. Each student was then provided a mindfulness bracelet and asked to wear it as much or as little as they desired. At the start of each survey, participants entered the last 4 digits of their phone number, which allowed for tracking of follow-up survey responses. Frequency of bracelet usage was assessed by each student who reported their percentage of bracelet usage from zero to 100 % in 50 % intervals.

The validated survey used to assess student affect was the PROMIS Positive Affect short form v1.0 survey, which is a 15-item survey used to assess positive or rewarding affective experiences, such as feelings and moods associated with pleasure, joy, elation, contentment, pride, affection, happiness, engagement, and excitement [14]. The instrument measures emotional affect over the past 7 days using a 5-point Likert Scale. The scoring of the survey was determined by summing the values of the response to each question. Student demographic information included race, ethnicity, and sex as well as medical school year, dual degree program enrollment, and specialty interest.

### Statistical analysis

Continuous variables are presented as mean +/− Standard Deviation (SD). Discrete variables are presented as N (%). The change of sum score over time was tested by using repeated measurement analysis. *P*-value < 0.05 was considered statistically significant. SPSS 27 (IBM Corp. Released 2020. IBM SPSS Statistics for Windows, Version 27.0. Armonk, NY: IBM Corp) was used for data analysis.

## Results

### Overview

Data collection began August 10th, 2020, with the administration of the PROMIS Positive Affect survey and collection of demographic information. The initial data collection included responses from 104 students (100% participation) followed by 78 students (75% participation) and 69 students (66.3% participation), respectively. All survey data was obtained via REDcap database created specifically for the purpose of this study, which allowed survey responses to be directly entered into the database by the participants via mobile device or laptop.

### Demographic data with baseline affect

Demographic data was well distributed among participants. Baseline affect, as measured by the PROMIS survey, was similar among male and female students. There was not a statistically significant difference of baseline emotional affect scores with respect to race, ethnicity, specialty interest, or enrollment in a dual degree program (Table 1).

**Table 1:** Demographic Information with Mean Sum Score at Baseline Participants’ characteristics

	Baseline Sum Score	
	Mean	Count
Gender		
Female	57.2	58
Male	57.6	44
Other	67.0	1
Race		
Black or African American	61.2	10
Asian	57.0	34
White	57.6	44
More than one race	54.7	12
Unknown	58.3	3
Ethnicity		
Hispanic or Latino	56.2	17
Not Hispanic or Latino	57.7	86
Specialty interest		
Primary care	58.5	11
Surgical	59.6	24
Non-surgical	56.3	22
Undecided	56.6	46
Enrolled in dual degree program?		
No	58.1	89
Yes	53.6	14

### Change in affect associated with frequency of mindfulness bracelet usage

**Fig. 1:**
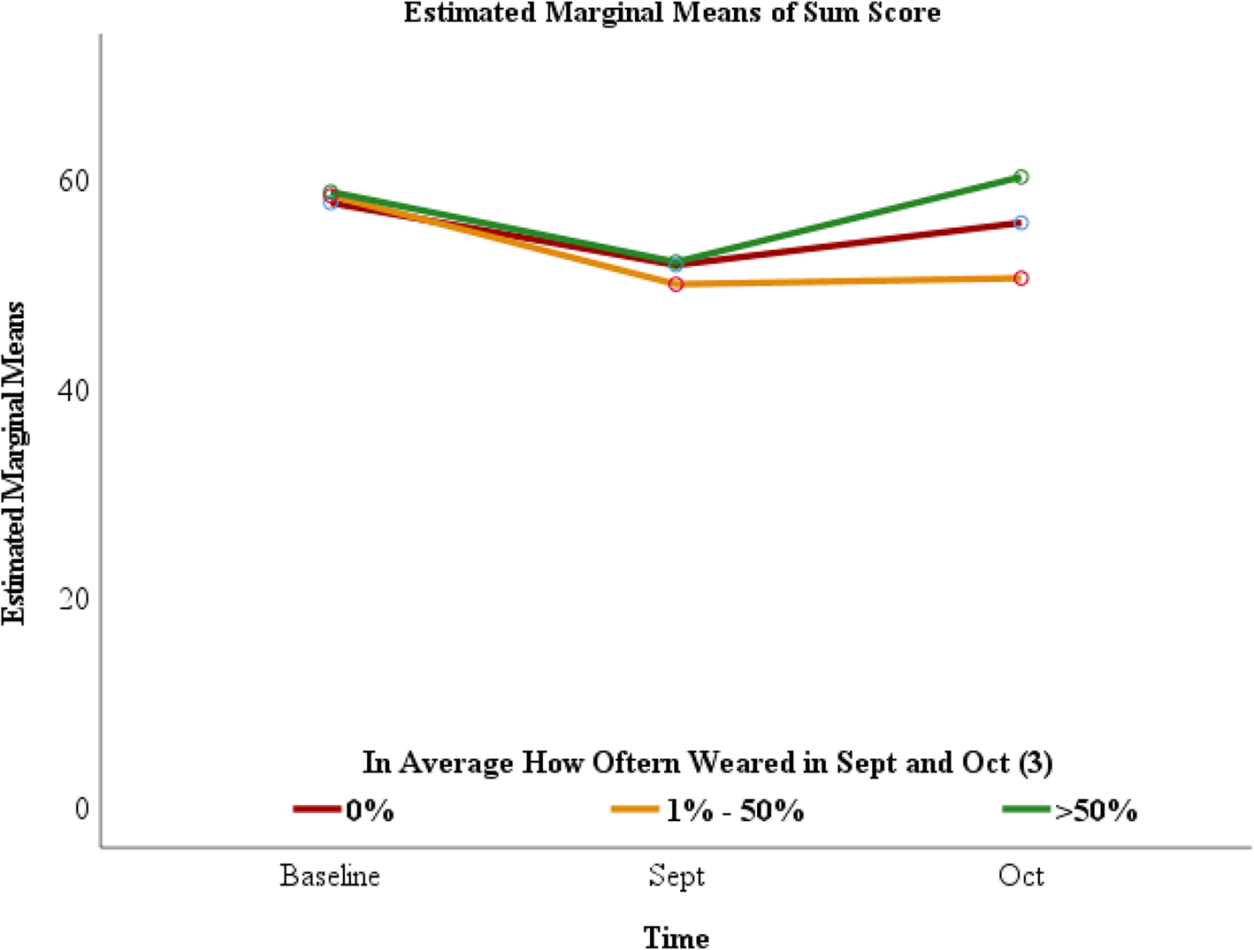
Change in Emotional Affect Sum Score Based on Mindfulness Bracelet Usage

Although positive affect over the two-month period was higher in students who reported wearing the mindfulness bracelet with greater frequency, the change over each month was not statistically significant (Fig. 1). The difference in sum score of emotional affect, as measured by the PROMIS survey was not statistically significant when comparing students who wore the mindfulness bracelet > 50% of the time to those who wore it 0% (*p* = 0.493) and 1 - 50% (*p* = 0.163) of the time (Table 2). Additionally, when comparing students who wore the bracelet 1 - 50% vs 0%, there was no statistical difference between emotional affect sum scores (*p* = 0.398) (Table 2), as depicted in Fig. 1.

**Table 2:** Difference of Sum Score According to Mean Frequency of wearing the Mindfulness Bracelet in September and October 2020

Sum Score in different time points according to the frequency of wearing the mindfulness bracelet					
How often wore the bracelet in Sept and Oct	Time	Mean	S.E.	95% Confidence Interval	
				Lower Bound	Upper Bound
0%	Baseline	57.7	1.72	54.30	61.18
	September	51.8	2.07	47.64	55.92
	October	55.8	2.21	51.40	60.25
1 - 50%	Baseline	58.4	1.89	54.63	62.21
	September	49.9	2.27	45.39	54.50
	October	50.5	2.43	45.66	55.39
> 50%	Baseline	58.8	2.20	54.37	63.20
	September	52.1	2.65	46.76	57.38
	October	60.2	2.83	54.54	65.89

*S.E.* Standard Error

### Change in emotional affect of medical students over time

At baseline, students reported a mean sum score of 58.23 on the PROMIS survey. Following 1 month of medical education, students’ positive affect significantly declined, regardless of mindfulness bracelet use (mean sum score 51.23; *p* < 0.01). During the second month of medical education, students’ emotional affect significantly improved (mean sum score 55.12; *p* < 0.01), but to a lesser degree than their baseline scores. In totality, sum score of positive affect was highest at baseline, declined in the first month, and then improved in the second month, albeit not to baseline levels (*p* = 0.02) (Table 3). All mentioned were statistically significant (*p* < 0.01).

**Table 3:** Mean sum scores of medical student’s emotional affect over 2 months

Sum Score in different time points				
Time	Mean	S.E.	95% Confidence Interval	
			Lower Bound	Upper Bound
Baseline	58.2	1.08	56.1	60.4
September	51.2	1.31	48.6	53.8
October	55.1	1.48	52.2	58.1

*S.E.* Standard Error

### Demographic factors associated with change in affect over time

**Fig. 2:**
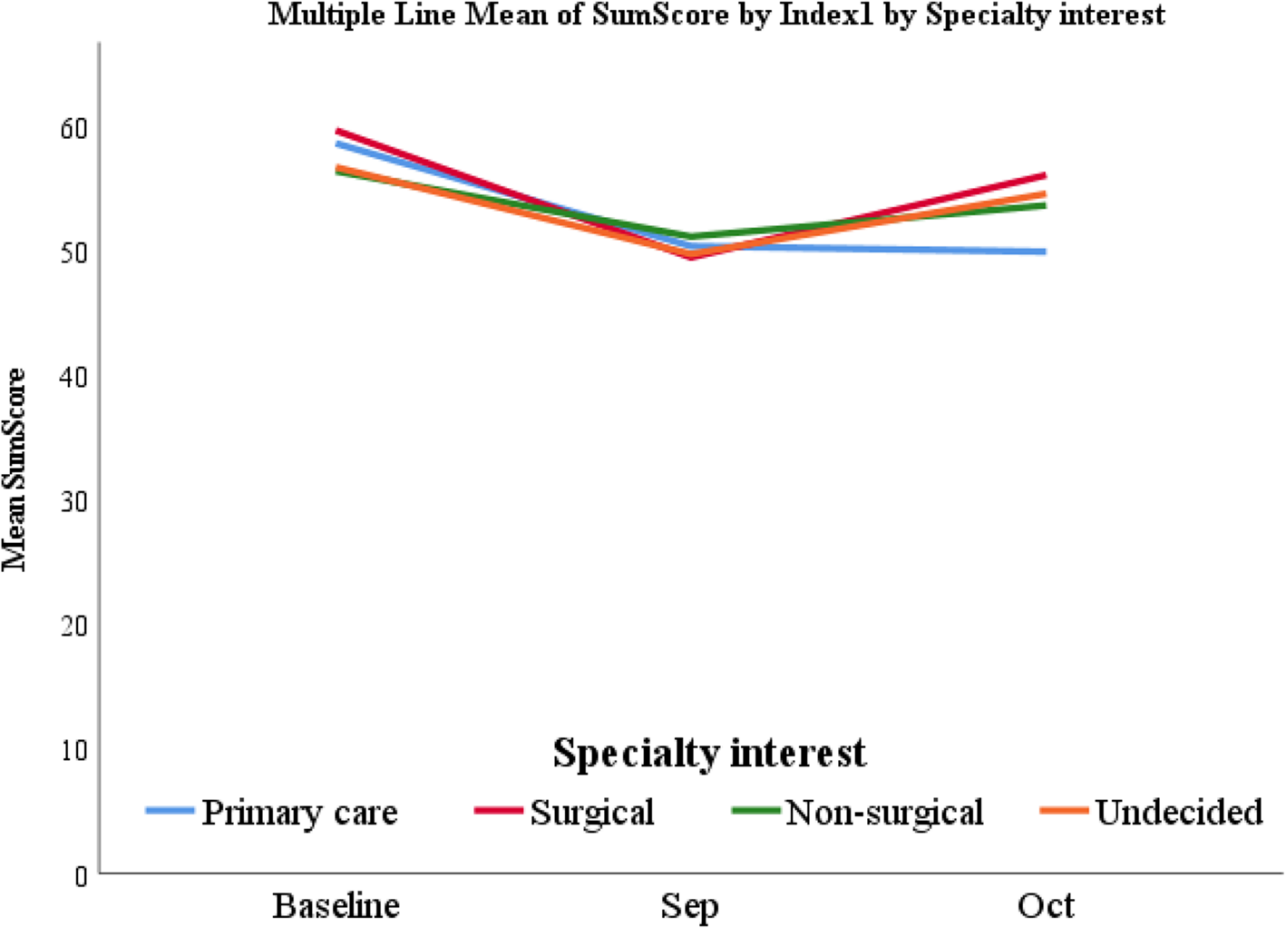
Change in Emotional Affect Sum Score Based on Specialty Interest

When reviewing demographic factors, we did not identify a statistically significant association with progression of emotional affect. The association of emotional affect based on the sum scores from the PROMIS short form survey with sex, ethnicity, and dual degree was not statistically significant (*p* values: 0.57, 0.46, 0.17, respectively). Additionally, specialty interest did not have a statistically significant association with emotional affect (*p* = 0.85) (Fig. 2).

## Discussion

This study examined the impact of a simple mindfulness bracelet on emotional affect. The mindfulness bracelet served as a reminder to practice informal mindfulness techniques introduced in a concise presentation. The study was delivered to a cohort of medical students known to undergo significant levels of psychological distress [8]. The psychological outcome measured was emotional affect. The brevity and cost-effectiveness of such an intervention without formalized mindfulness training provides a novel opportunity to combat the known psychological distress that occurs in medical students [15].

Although we did not identify significant improvement in emotional affect with mindfulness bracelet usage, the positive trend in emotional affect identified with increased usage is promising. This positive trend is consistent with previous studies that demonstrated an improvement in perceived stress and overall mental health with the use of informal mindfulness practice [8]. Additional studies observing other simple mindfulness modalities such as the Calm app have shown to be effective modalities to reduce stress and improve self-compassion in undergraduate students [16]. These results are consistent with mindfulness cues and techniques such as studies including the use of mindfulness reminders on the Apple Watch [16, 17], as well as diaphragmatic breathing on stress [18]. Our modest findings as well as previous studies justify further exploration of simple, inexpensive, and time-efficient interventions such as the mindfulness bracelet.

This study found that medical students’ affect worsened over the first month of medical school, before partially recovering the following month. This is consistent with previous studies which have found level of training to be correlated with increased psychological distress and decreased empathy [4]. The overall decline in scores from baseline could be due to the stress associated with the transition to medical school expectations [7, 19, 20]. These include the adjustment to the medical school environment, increased scholastic workload [21], interpersonal interactions between learners and teachers [22], and/or exposure to the clinical environment [2]. The improvement in student affect from the first month to the second month of school has previously been explained by students’ adjustment to medical education including better study habits, the balancing of academic and personal obligations, the establishment of friendships with fellow colleagues, and the implementation of coping strategies to adapt to stress [2, 21]. Strategies that involve engagement, including reliance on social support, expression of emotion, and implementation of mindfulness practices lead to adaptation which can reduce anxiety and their effects on mental and physical health [13, 23].

While not all demographic groups in our study demonstrated the same recovery in emotional affect over time, we did not discover a statistically significant association between demographic data and progression of emotional affect over a 2-month period. Though the distribution of ethnicity and gender within programs and the intrinsic and extrinsic biases certain demographic groups may face has previously been associated with worsening emotional outlook in medical students [5], our study did not replicate these findings. Identifying specific factors that may affect the emotional transition of students from various demographic groups to medical school, including the physical learning and working environment, access to counseling, and peer and faculty interactions may provide further understanding. Additionally, dual degree students (i.e. PhD, MBA, or MPH) have been associated with increased stress secondary to academic and professional pressures and increased scholastic workload [7], which was not statistically significant in our study. Our negative findings with regards to the association of demographic factors are inconsistent with previous literature and may be a product of the medical environment that exists at the institution studied. This inconsistency justifies further investigation to better understand which factors within a medical school environment lead to an equitable emotional transition among different sexual orientations, ethnicities, and professional tracks.

This study has several limitations. First, confounding variables may affect the results found in the study including academic performance, socioeconomic factors, and personal events that may limit the survey results. Additionally, it is difficult to understand the explanations for the trends identified in the study without qualitative data to draw from. The study was also performed at a single medical school and within one class year, and therefore may have poor external validity. Lastly, the study is limited by the fact that three surveys were collected over a two-month period. More longitudinal data may reveal additional trends; however, there was an increasing number of non-responders whose bracelet usage is unaccounted for. The decreasing participation rates observed in this study highlight one of the major challenges when attempting to obtain longitudinal data.

## Conclusions

This study provides interesting and significant results that support further investigation. Additional research aimed at understanding and modifying the early decrease in emotional affect, particularly given that student affect did not recover to its baseline, is critical to medical education. Increasing the awareness and importance of mental health in the medical student population continues to be an important topic. More investigation is needed to explore alternative interventions that will improve the overall mental health of our future physicians.

## Data Availability

The datasets used and analyzed during the current study are available from the corresponding author on reasonable request.

## Abbreviations

PROMIS: Patient Reported Outcomes Measurement Information System
IRB: Institutional Review Board
SD: Standard Deviation
